# A Qualitative Study on Parental Experiences with Genetic Counseling After a Positive Newborn Screen for Recently Added Conditions on the Recommended Uniform Screening Panel (RUSP)

**DOI:** 10.3390/ijns11040101

**Published:** 2025-10-30

**Authors:** Macie Hricovec, Amy Gaviglio, Christina Mealwitz, Michelle Merrill, Aaron J. Goldenberg

**Affiliations:** 1 Department of Genetics and Genome Sciences, Case Western Reserve University School of Medicine, Cleveland, OH 44106, USA; mcm9@case.edu (M.M.);; 2 Connetics Consulting, LLC., Minneapolis, MN 55417, USA; 3 Licensed and Board-Certified Genetic Counselor, Columbus, OH 43214, USA; 4 Department of Bioethics, Case Western Reserve University School of Medicine, Cleveland, OH 44106, USA

**Keywords:** newborn screening, genetic counseling, genetic diseases, parental experiences, X-linked adrenoleukodystrophy, X-ALD, Pompe disease, spinal muscular atrophy, SMA, rare disease

## Abstract

The goal of newborn screening (NBS) has remained the same despite its significant expansion from its inception as a public health initiative. This goal is to identify infants that are at risk for a set list of conditions and to implement a care plan to prevent, delay, or mitigate adverse health outcomes for those affected. The role of genetic counselors (GCs) in the NBS space is currently evolving, and there is limited research on parental experiences with genetic counseling for more recently added conditions on a list approved by the U.S. Secretary of Health and Human Services called the Recommended Uniform Screening Panel (RUSP). This qualitative study interviewed parents who have spoken to a genetic counselor after their child was diagnosed with one of three following conditions in the past five years: Pompe disease, X-linked Adrenoleukodystrophy, and Spinal Muscular Atrophy. A total of 13 interviews were conducted and results were organized into five thematic areas: (1) NBS/Results Disclosure, (2) Diagnostic Process after NBS, (3) Treatment/Follow-Up, (4) Communication, and (5) Holistic Support. The findings of this study highlighted parental preferences for early involvement of genetic counselors, provider, and parent education on NBS, and the provision of family support beyond genetic resources.

## 1. Introduction

Newborn screening (NBS) is a widely recognized public health service that aims to identify infants with conditions included on the screening panel and implement a treatment plan prior to the presentation of symptoms. This nationwide and state/territory-run program began in the 1960s, and since then has expanded in the number and types of conditions each newborn is screened for as technology, including tandem mass spectrometry and next generation sequencing (NGS), has progressed [1]. With many advancements in NBS technology comes the need for individuals in this space to effectively communicate screening results and navigate the uncertainties that arise with providers and families.

While each state and territory have the ability to include or exclude conditions on their screening panel, the Recommended Uniform Screening Panel (RUSP) serves as a guide. Included on this recommended list of conditions are a set of core conditions, which have tests available, recognized health outcomes, and available treatments, and a set of secondary conditions, which are incidental findings from core condition screening [2]. From the first condition, phenylketonuria, added in the 1960s to 38 core conditions on the RUSP in current day, the amount and diversity of conditions being screened across states’ panels has expanded and along with this, so has the amount of information parents are receiving after their child is born. Conditions that were previously diagnosed after the onset of symptoms or through known family history are now being diagnosed after out-of-range, or screen-positive, newborn screen results. With this expansion in screening and increase in available information, the involvement of genetic counselors in navigating families through an abnormal newborn screen for the genetic conditions included on the panel is critical. Genetic counselors in the newborn screening space serve as an important source of support and education for parents while also helping families roadmap the best medical care for their child [3].

Recently added conditions on the RUSP include X-linked Adrenoleukodystrophy (X-ALD), Glycogen Storage Disease Type II (Pompe disease), and Spinal Muscular Atrophy (SMA). All three of these conditions have been added within the last 10 years but not all have been implemented into every states’ NBS panel. The following is a brief description of the three conditions this study will be focused on and the unique genetic counseling issues they present.

### 1.1. Pompe Disease

Glycogen Storage Disease Type II, or Pompe dieseae, was added to the RUSP in 2015 and is currently screened for in 44 states [4]. Pompe disease has an autosomal recessive inheritance pattern, and the current first-tier test available measures acid alpha-glucosidase (GAA) enzyme levels in the newborn’s blood; additional testing, such as genetic testing with blood sample and an echocardiogram, needs to done to confirm a diagnosis [5]. Unique genetic counseling issues for families with a newborn that has an out-of-range GAA result include false-positives involving pseudodeficiency alleles and the different types of the condition: classic infantile-onset, non-classic infantile-onset, and later-onset [5]. The variability between types in onset and severity of Pompe disease leads to complex conversations about prognosis and when to begin treatment, such as enzyme replacement therapy (ERT) [6].

### 1.2. X-Linked Adrenoleukodystrophy

X-ALD is currently being screened for in 38 states since being added to the RUSP in 2016 [7]. This condition, as the name suggests, has an X-linked inheritance pattern of a pathogenic variant in the *ABCD1* gene and primarily involves the nervous system and adrenal gland independently [8]. A positive newborn screen for X-ALD involves an elevated C26:0-LPC level in the dried blood spot and is confirmed via molecular testing of the *ABCD1* gene [9]. While males are typically more severely affected with X-ALD, both males and females can screen positive for this condition on NBS; this adds a unique issue for genetic counseling as there are different approaches and next steps depending on if a male or female screens positive [9], which could potentially make the parental experiences with genetic counseling different based on the biologic sex of the child that screened positive in a family. In addition to male verse female positive screen protocols and experiences, other genetic counseling issues that may impact parental experiences and preferences are the lack of genotype-phenotype correlations, the need to test other at-risk male family members, the reported 4% de novo rate, and the immediate referral to multiple specialty providers for newborn males identified on NBS with confirmed *ABCD1* pathogenic variants [8].

### 1.3. Spinal Muscular Atrophy

SMA was added to the RUSP in 2018 and the number of states in the U.S. that are currently screening for this condition is 48 [10], making it the most recently added and the most widely screened out of the three conditions included in this study. This condition has an autosomal recessive inheritance pattern, with a 2% de novo rate [11]. Because this condition’s carrier frequency may be as high as 1:40 to 1:60 depending on patient-reported ancestry or ethnicity, The American College of Obstetricians and Gynecologists (ACOG) recommends that carrier screening for SMA be offered to all women who are pregnant or considering becoming pregnant [12,13]. While carrier screening and newborn screening are two separate screening tests, they have intersecting goals, with both aiming to better assess risk. In the case of SMA, carrier screening identifies a couple’s risk of having a child with SMA while newborn screening identifies a child at risk of SMA. Included in SMA’s clinical characteristics are atrophy and muscle weakness due to degeneration and loss of anterior horn cells, with a variable onset of muscle weakness ranging from birth to adulthood [11]. The recent advances in gene therapy and treatment for SMA have changed the outlook of the diagnosis for some families [14]. Unique issues for genetic counseling and parental experiences with SMA include prior conversations about the condition in a prenatal setting and how this impacts parental preferences, new targeted treatments, and the interactions between the *SMN1* and *SMN2* genes that impact SMA phenotypes and severity of the condition.

Although the goals of identifying conditions through newborn screening are clear, the opinions and parental preferences surrounding timing of speaking with a genetic counselor is understudied. Previous studies have looked at parents’ experiences with uncertainty during their diagnostic journey after a positive newborn screen and have echoed the importance of healthcare providers meeting the needs of this parent population [15]. Another study published in 2024 discussed the parental experiences of genetic counseling with parents of patients diagnosed with Spinal Muscular Atrophy (SMA) through NBS as well as parents of patients diagnosed clinically and found that most, but not all, parents found it helpful to meet with a genetic counselor shortly after the diagnosis was made; however, this study also shared parents’ experience of an overwhelming first appointment [16]. These findings emphasize the need to explore parental experiences and preferences when being referred to genetic counseling in order to ensure the most relevant and helpful information is being shared at a time when it is both useful and digestible. Genetic counselors in the newborn screening space serve as an important source of support and education for parents while also helping families roadmap the best medical care for their child [3]. There are opportunities for genetic counselors to mitigate some of the parental anxiety and uncertainty families experience during this vulnerable time, such as providing psychosocial support, up-to-date information, and accessible patient friendly resources.

## 2. Materials and Methods

### 2.1. Study Design

The approach of this research was a qualitative study using semi-structured interviews. This study was approved by the Institutional Review Board (IRB) of Case Western Reserve University and conducted as part of the primary researcher’s (MH) graduate student research project.

### 2.2. Participant Recruitment

Participants were recruited through advocacy organizations, including direct outreach by parent advocacy organization leadership to families, social media posts on Facebook and Instagram, and information shared about the study on the organization’s website. The three advocacy organizations participants were recruited from were Pompe Warrior Foundation, ALD Alliance, and CureSMA. Information shared with advocacy organizations included an infographic detailing the study and both a QR code and web link to the screening survey created with the platform, Qualtrics.

### 2.3. Screening Survey

The screening survey verified the participant was (1) a parent of a child diagnosed with one of the three RUSP conditions: Pompe disease, X-ALD, SMA, (2) the child was identified through NBS, and (3) the parent had met with a genetic counselor regarding their child’s diagnosis within the past 5 years. If eligibility requirements were met, a brief set of three demographic questions were asked on the following page of the Qualtrics screening survey. These questions asked about the participant’s highest level of education completed, total household income, and ethnicity.

Additionally, the interested participant’s first or preferred name, child(ren)’s first or preferred name(s), and parent’s preferred email were requested. Sharing the first/preferred names of the parents and children in the screening was optional but encouraged, as a way to personalize the interview session. The email address was used to contact interested participants to arrange a time for a Zoom interview as well as share the consent document and list of questions from the interview guide. This allowed the participants time to look over the documents prior to the Zoom call.

### 2.4. Semi-Structured Interviews

A semi-structured interview guide was drafted by the primary researcher (MH) and underwent multiple revisions prior to and after the guide was piloted. A pilot interview was conducted with a researcher who has worked on previous projects focused on parental experiences with newborn screening. Interviews with study participants were conducted between October 2024 and January 2025. The primary researcher (MH) conducted all interviews, and all participants were made aware that MH was conducting this research to fulfil her master’s degree requirements. Length of the interviews ranged from 23 min to 79 min, with an average length of 46 min. All interviews were conducted via Zoom.

### 2.5. Data Analysis

Zoom platform’s autogenerated transcript feature was used to create the initial transcript for each recorded interview. The audio recordings and transcripts were stored securely in Box. The initial Zoom transcript was then reviewed by the primary student researcher and edited to ensure accuracy. Each interview transcript was assigned a number along with a letter indicating which RUSP condition the parent’s child had been diagnosed with as a reference code. Once each transcript received its reference code, the transcripts were deidentified and the audio recordings were deleted. The interviews were coded using the Dedoose (version 9.2.22) qualitative analysis software. A draft codebook was developed by the student researcher with collaboration from an experienced qualitative researcher. The codebook was repeatedly tested and modified before being used with all transcripts. The student researcher coded all 13 transcripts, while a second coder evaluated 20% of the transcripts. Using grounded theory analysis, common themes were extracted within and across participant comments to draw conclusions with respect to the study aims. Generally, we reached saturation within our sample across all themes.

## 3. Results

A total of 17 individuals completed the screening survey and all 17 screening surveys met eligibility. Out of the 17 respondents that were contacted via email with follow-up information, 13 participants scheduled and completed a Zoom interview. Table 1 includes the demographic information collected from the 13 participants, including 5 parents from the X-ALD community, 7 parents from the Pompe community, and 1 parent from the SMA community. While not information directly collected through the screening survey, all participants in the survey were the mothers of a children that screened positive on newborn screening.

During interviews, two parents shared that they had not spoken to a genetic counselor; one participant was not aware of the differences between a genetic counselor and a geneticist, and one participant went to a genetic office where a nurse practitioner took on some of the same roles as a genetic counselor. While both participants had not spoken to a genetic counselor, their interviews were included in this study as the experiences shared added to the themes identified in this study.

Five primary thematic areas were identified in the data of the interviews, outlined with subthemes in Table 2. Three themes represent the different stages in the timeframe of the newborn screening experience that impact a parent’s perceived utility of speaking to a genetic counselor and the information the family is seeking. These themes include newborn screening/results disclosure, diagnostic process after NBS, and treatment/follow-up. In addition to these three themes related to the timing, there were two additional themes that overlapped different stages of time in a parent’s journey: communication and holistic support after NBS. Additional representative quotes from parent interviews can be found in Table S1 of the Supplementary Materials section.

Newborn Screening/Results Disclosure

In this theme, participants detailed the impact that their family’s newborn screening process and result disclosure had on their family’s experience.

### 3.1. NBS Workflow

Multiple families shared how issues within the newborn screening workflow delayed results disclosure and confirmatory testing. One parent shared her experience doing multiple blood draws after inconclusive results without knowledge of what was flagging on the newborn screen:

*“The results are inconclusive. And you know, we need to test again. [the midwife]’s like, maybe I didn’t get enough blood or something the 1st time…then I get a call from the pediatrician at this point. Because we went through the pediatrician after like the couple of weeks with the midwife. And they’re like, yep, it’s inconclusive again, we want you to go to a local lab like in your area. And he needs to give blood again…Never once was the word adrenoleukodystrophy, ALD even brought up to me…they were not telling me that something’s flagging here… it took a solid like 3 to 4 months before I found out we’re dealing with adrenoleukodystrophy here.”*

*(A1)*

Another parent shared that her child’s NBS results had gotten lost in the mail, delaying the time at which the results were communicated with her family:

*“She was…a little over 2 weeks or so when we got the phone call…the only reason, like I found out that it got lost in the mail was because I had seen that, like a lot of other people with the newborn screen got their results within like 5 or 7 days. And so I was wondering, I was like, why was hers so late? And when I asked that, that’s when the genetic counselor told me that they were actually lost in the mail.” *

*(B4)*

The way results are communicated with providers and families can vary state to state. The provider collecting the sample and receiving the results also differs depending on the setting in which the child was born, such as a hospital birth versus a home birth. Families’ experiences emphasized the impact that potential logistical errors in the newborn screening workflow can have on the results disclosure and timing of referral to genetic counseling.

### 3.2. Parent’s Knowledge of NBS

A majority of participants shared that they wished they had more knowledge on newborn screening prior to receiving the result. A few participants stated that they were familiar with a heelprick being done on newborns shortly after birth but were not fully informed on what the collected blood was used for.

*“You don’t really realize what they’re doing in the hospital like that…It’s so abstract…even if a nurse would have sat down and said, even for like 5 min, and said, What we’re testing for this is why, you know, and just, you know, even just to have a little bit of a primer, I think, would have been helpful.” *

*(B7)*

Other participants that did receive a brief overview of what newborn screening is felt like the explanation of what happens if something comes back positive on the screening was not mentioned. These participants echoed that the conversation was over-reassuring, with providers communicating that this is not something parents will have to worry about.

*“They kind of just like whisk your baby away in the hospital. They tell you briefly, the hospital staff, like what it’s for. You know, they say if something’s wrong you’ll get a phone call. But don’t worry, you’re not going to get a phone call like, very just kind of like this never happens… And also they kind of glossed over it like we didn’t have to worry, and it ended up we really did. So I wish there was just more education around it, initially, like what it is.” *

*(A5)*

The parents’ knowledge about newborn screening varied from conversations with providers about the screen prior to their child’s birth to conversations after birth to no conversations at all, leading to a spectrum of understanding. However, a common theme with a majority of participants in this study was that they wanted more information and education about newborn screening prior to their child’s NBS results.

### 3.3. Impact of Results Disclosure

Parental experience receiving the screening results was different for each family, with most participants in this study receiving the news via phone or a video call. Other factors varied between participants, including which parent received the phone call, what type of provider shared the results, and the information that was shared during the result disclosure. Common themes included receiving outdated information about the condition, a primary care/non-genetics provider sharing the results, and the provider to avoid Googling the condition. Parents’ experiences with the results disclosure impacted their perceived utility and preferred timing of speaking to a genetic counselor or genetics provider.

Information shared during the results disclosure depended on the provider sharing the results. Many participants in the study shared that the explanation of the condition’s prognosis and lack of opportunities to have questions answered at the time of the results disclosure negatively impacted their experience.

*“She’s been a doctor for 40 years, has never seen a case like this before, that it’s associated with heart issues, that there’s no surgery. There’s nothing that you can do to treat it. And so I was like, I don’t like, what are you saying? And she said, I’m sorry. That’s how the conversation was left…So that was completely different, obviously, like knowing about Pompe now…it’s not a death sentence. And so for her to like, know nothing about the disease, and then, like act like she did, was kind of traumatizing to be honest.” *

*(B4)*

*“I got a phone call…from an LPN at the pediatrician office, and her words to me were, ‘Listen, I don’t know what this is, but don’t Google it. [Child]’s newborn screen came back for something called X-ALD. I can’t even tell you what it is’. The pediatrician was [out of town]. So there was nobody to tell me what it was, nobody to explain to me what it was.” *

*(A2)*

A participant that received the results disclosure call from the genetics office shared:

*“They were like, you know, we just were wanting to get in touch with you to let you know that your son {child} did screen positive for Pompe disease. But that’s not a confirmatory result. That just means that on his heel prick test in the hospital, he has low enzyme activity…I will give her a lot of credit. She did a really good job of trying to comfort husband and I, and in the sense of like—I was able to ask, you know, could it be a false positive? And, she explained, kind of that those are very common because of the way that the newborn screeners are done.” *

*(B6)*

Participants preferences for an ideal results disclosure included having a specialist or genetic counselor sharing the results instead of the pediatrician. Multiple participants shared that a major component of wanting a specialist or genetic counselor involved in the results disclosure was the desire to ask questions and get knowledgable answers.

*“I think introducing yourselves [genetic counselors] and having you guys [genetic counselors] on either the same phone call and or having us physically come in before you break that news…we would have loved to have that.” *

*(B2)*

*“I think in an ideal world it would be either the specialist or the genetic counselor calling us first, and not even having the pediatrician share that…I know it seems like a very common theme that pediatricians share the news…in my mind, because when you have questions and it’s someone calling you who can’t answer those questions, it’s a little awkward.” *

*(A4)*

While many parents expressed wanting the opportunity to ask questions, some participants did share that due to the shock of the results disclosure and uncertainty at this point in the diagnostic journey, not all parents had questions at the time that the initial screening results were being shared.

*“I think I was so kind of shocked that I didn’t even think to really ask any questions. I don’t even think I knew what to ask.” *

*(B7)*

*“So yeah, I guess we could ask questions, but it was still a lot of unknowns. We weren’t, you know, we couldn’t be satisfied with anything, because there was no, not enough information.” *

*(A5)*

A common theme in almost every interview was the parent being advised during the results disclosure not to Google the condition. There were differing parental reactions to the Google results. One participant stated that she did not go on Google after the results disclosure; however, majority of participants in this study did Google the condition, expressing that they wanted more information then what they were given during the results disclosure. This suggests that even with warnings from providers about Google searches, the parents’ desire for additional information often outweighed the potential psychological harms associated with researching the condition on the internet prior to confirmatory testing.

*“The worst part of it is the suspense…the first thing they’ll tell you is, ‘don’t go Google this.’ Well then everybody’s gonna go Google, that and so you end up with just this horror of what you see on Google from 15 years ago, when children with infantile disease were sent to hospice…that’s every parent’s worst nightmare.” *

*(B6)*

Overall, the experiences of receiving the newborn screening result and the information shared by the provider varied from parent to parent and reflected the parents’ motivation and sense of urgency for speaking with a genetic counselor or genetics provider.

Diagnostic Process after NBS/Prognostic Journey

Parents in this study shared their family’s experience scheduling a genetic counseling or genetics appointment after receiving the newborn screen result. Most parents expressed wanting earlier appointments as a way to mitigate their stress or panic from the results disclosure. Participants also shared what information was helpful, such as treatment information and balanced approach when providers are explaining the condition’s spectrum of presentation, as well as what information was unhelpful, including focusing on the severe end of a condition’s spectrum or using complex medical jargon.

### 3.4. Scheduling/Timing of Initial Genetic Counseling Appointment

When the initial genetic counseling appointment was scheduled and what that scheduling process looked like was different for each family, with some parents sharing that they had the ability to pick the time and other parents sharing that an appointment was scheduled for them. Parent preference for options in the timing also varied, with some parents wishing they had more control of the timing and others sharing that they appreciated the scheduling being done on their behalf.

*“The soonest appointment any place gave us, we made work.” *

*(B3)*

*“We were not given any options. I was appreciative that the appointments were made for us because, given the potential severity of the condition, we would have went at any time. But because I had just given birth, I was off of work. My husband is essentially self employed, so he was able to step away from work and go with me to that appointment. At the time we had a really strong support system within our family, you know, grandparents. And so that was very helpful.” *

*(B4)*

Factors that influenced the timing of the genetic counseling appointment that were family-dependent included travel to clinic, time off of work, and vacation plans. Even though most participants shared that maternity leave allowed them some flexibility, there were still some considerations of work and coordination for this initial appointment.

*“It was absolutely more on our end…like traveling, our jobs. And you know, I was on maternity leave. My husband was on maternity leave, but we still were kind of keeping an ear to the ground work wise. So there were just a lot of different considerations.” *

*(B6)*

In terms of factors that were provider- or clinic-dependent, participants shared limited availability and days when the genetic clinic had GC appointments as well as if the clinic provided options and/or parent involvement in the scheduling process.

*“I called the hospital where the genetic counselor was that moment, and was like, I need someone to explain to me what’s going on. Can I please come in now? And they told me no, nobody could talk to me…I had to wait until my appointment on Monday, that they only did genetic counseling on Mondays, and it was like a Wednesday or something.” *

*(A2)*

*“The genetic counselor set up the appointment and everything. So when my pediatrician called, she said,… you’re going to city] on Wednesday at 10 a.m. So they had set up everything prior, so I didn’t have to do any of that.” *

*(B4)*

Other factors outside of the provider and the families’ control included the time of year and delays in newborn screening results.

*“And we were leading right into Christmas…the rush was to get [child] seen before the holiday…so I’m grateful that they were able to push everything through. But then we kind of had to hit pause, because, you know, the genetics clinic was going on vacation, and you know, with the New Year and the holiday. Everything became very spotty.” *

*(B1)*

Varying experiences with scheduling the genetic counseling appointment emphasize the many factors that impact provider and family availability. Majority of parents interviewed in this study expressed wanting to speak with a genetic counselor as soon as possible, recognizing the impact that information shared during the results disclosure and perceived urgency of a diagnosis had on levels of parental panic as well as implementing a care plan for their child.

### 3.5. Initial Genetic Counseling Appointment

Information that families found most helpful was different for each participant, with variability depending on if the parent had other children at risk, familiarity with the condition, prior information shared by other providers, and what the family’s expectations were for the appointment. There were many parents that shared that they appreciated printed out information discussed at the appointment as well as additional resources with accurate and up-to-date information that the family could refer to.

*“But then she hands us this folder, and the folder had every single piece of paper that she had talked about…for us to take home to read over. And I can’t tell you how much of a comfort that was for the next couple of days, maybe week.” *

*(B2)*

*“She just gave me different websites to go on to as well, but I think that would be something helpful again, just to give parents more information that’s accurate instead of inaccurate stuff.” *

*(B4)*

Parents also noted that they appreciated an open and honest conversation with the genetics providers that created a space where parents’ questions could be asked and information was not “sugar-coated.”

*“They were both good about saying there’s like no dumb questions. Ask like, literally anything that pops into your head, even if you think it’s silly.” *

*(C1)*

*“She’s just very patient with us, and answered all of our questions…she’s honest with us…they physically examined [child], she was like, ‘she looks great. Her liver enzymes are normal…she’s healthy right now, and that’s great. That’s a good sign’…once we did finally meet with them, it was very helpful. I just wish we got a little dose of that sooner.” *

*(A5)*

Information that was noted as helpful in the initial genetic counseling appointment was disease outlook and additional information about how the disease works. Many parents shared that hearing about how other individuals with the condition are doing provided a sense of hope.

*“They also shared…in that first appointment about some adult patients they had with Pompe, and even just hearing, like real life stuff was really helpful…I remember them saying, we have an…adult patient with Pompe that’s a lawyer… even just that, those pieces again, some kind of anecdotal stuff was really nice.” *

*(B7)*

In regard to information about risk to other family members or recurrence risk for future pregnancies, some parents found this information helpful in the initial genetic counseling appointment and other parents found discussing this to be overwhelming.

*“She said she could tell us more about like the options if we wanted to have more kids, and what the odds of a future kid having it would be, and how to handle all of that, and like obviously being like 5 days postpartum, I was not in any place to be thinking about that, but it was good to know that, like she was there as a resource.” *

*(C1)*

*“They did do a good explanation of like the pure genetics behind it…And then, I think the education on the likelihood of older brother having it, I think was also really helpful. Because once I heard that it was a only a 25% chance that he would also have it too, [I was] able to breathe a little bit more.” *

*(B7)*

In addition to information that parents found helpful, participants were also asked if there was information presented at the initial genetic counseling appointment that was not helpful. While some parents found condition outlook and mentions of other individuals with the same diagnosis helpful and hopeful, some parents shared the opposite response when it came to provider sharing individuals that were on the other end of the spectrum. Participants in the study noted the impact that only sharing one side of the spectrum of the condition had on their perception of the outlook of their child’s life.

*“He’s like I, in fact, had a patient who recently died from this. And so I’m in panic mode at this point, like I’m scared for my son’s life.” *

*(A1)*

*“My biggest beef and my biggest gripe, with how they explain the condition is that they don’t give you any sense of hope. I left that call, thinking my child would be lucky if he saw the age of 8. They never told us that he could live an asymptomatic life. They never told us that there was a life that he could live with just even Addison’s disease….there was never ‘you could live an asymptomatic life’…They explain the condition to us and basically leave us thinking like our son is going to slip into a vegetative state and die if he’s lucky by the age of 8.” *

*(A3)*

Another parent frustration was information being presented at a higher level than what the parent could understand, such as science or testing technology.

*“Being able to explain, like the tests a little bit like in terms that people who don’t do this every day would understand…I felt like I had to keep asking like, ’all right, so this test is testing for this. What does that mean?’…There’s just so many tests and labs and stuff that they’re looking at, that someone who’s never done that, it’s overwhelming hearing it.” *

*(B4)*

*“He talked to me like I was on his level. But I wasn’t. I’m not a geneticist. So you’re like hitting me with like XY chromosome. And here’s this, this, this, this like. It’s probably why I don’t remember it because I’m like, what like, I don’t know what you’re talking about. I don’t understand genetics.” *

*(A1)*

During the initial genetic counseling appointment, parents recognized that there is still a lot of uncertainty around the diagnosis, such as true positive vs. false positive, carrier vs. affected, or infantile-onset vs. late-onset. Numerous parents noted that information shared regarding these uncertainties was unhelpful and in some cases created false hope.

*“[The genetic counselor] had reassured me at that time that it was not possible that he was going to have infantile Pompe… that’s what it ended up being is that he does have an infantile variant and a late onset variant, and then I know at that time she had reassured us to like, based on the one common variant that he had, most people don’t start treatment until way later in life… I would much rather have you tell me you don’t know than try, and just, you know, Fake it till you make it.” *

*(B5)*

*“When we got to the genetic counselor, she was very nice. She was very informative, but she did give us a little bit of false hope because she told us that she had worked in [state] previously when they had just implemented the newborn screening and that there were lots of false positives. especially when the very beginning.” *

*(A2)*

Participants emphasized the need for digestible, accurate and straightforward information during the initial genetic counseling session. Unhelpful information varied from parent to parent but there were recurring comments of unbalanced presentation of the condition’s spectrum as well as speculated outlook prior to diagnostic genetic testing.

### 3.6. First Family’s Experience

Multiple families within this study were the first family in their state to have a child screen positive on newborn screening for a specific condition. Parents shared the impact that this had on their experience while also acknowledging the complexity.

*“We had to give a lot of grace to the providers, and people that gave us this information because they just they didn’t know how to how to relay the information that she was positive in a in a way that parents should receive this information…It was a learning curve for everybody involved.” *

*(B2)*

In addition to parents sharing the impact that being the first family navigating a positive newborn screen for a condition in their state had, participants also shared the impact that being the first family within their family to receive this diagnosis had. Participants expressed the weight and responsibility they felt to share their child’s diagnosis with other family members and inform extended relatives of their risk.

*“Any first family, it’s hard. It’s really hard. And I think those first families are the ones that probably need more support…If any of my cousins were to have kids who came back on newborn screening, it probably wouldn’t be as traumatic on them. I think the combination of being postpartum and being a first family is just overwhelming.” *

*(A3)*

*“You think like, oh everyone’s gonna be so happy we’re sharing this…I don’t want someone else getting pregnant and like finding this out and coming back to tell us. And we’re like, oh, yeah, we knew, right?…And so I think we felt like we’re doing the right thing by sharing it with the family members that needed to know. But yeah, when it’s received differently…it does make it hard. Family takes it all in different ways…It’s kind of my experience like no one was as concerned about it as I was.” *

*(A4)*

Participants being the first family in their state or within their family contributed to their experience within the diagnostic period after their child’s newborn screen result and emphasized the importance of supporting first families in their journeys.

Treatment/Follow-Up

### 3.7. Testing Family Members

Many participants shared the desire to have family members, including parents and siblings, tested sooner rather than later. While there were differences between conditions in urgency of testing siblings, there was a common theme of parental concern for older siblings that would not have been screened for the condition at the time they were born.

*“But I have a son who was 3 at the time, and the newborn screen for ALD didn’t exist when he was born. So it was like this realization that [my son] had never been screened, and there was a good chance that he also had it. And we didn’t know. And it was just so many questions and so much panic” *

*(A2)*

*“And then we had to get [oldest child] tested because newborn screening was only permanently on the [state] screen for about 6 months before [youngest child] was born. So then we had to wait a whole other month to get [oldest child’s] results to see if he had Pompe as well…I don’t know which was more stressful.” *

*(B7)*

Throughout the interviews, there were differences in protocols of testing family members between the three conditions, such as X-linked ALD and autosomal recessive Pompe, as well as differences in experiences with cascade testing between family’s same diagnosis. The following are two different parent experiences with testing older siblings for Pompe:

*“One thing for our main genetic counselor that I really appreciated was she was very proactive…If you have a confirmed diagnosis for a condition there, I think it’s Invitae that offers free testing for siblings…and so she got all of that set up for us very quickly, and there was just a lot of forethought put into how she handled our case, which I really appreciated.” *

*(B1)*

*“Our genetic place hasn’t let us [do] like the full genetic workup on [child’s siblings]. So we did do just like enzyme levels to determine whether or not they would have had it.” *

*(B5)*

Participants’ experiences with testing other family members varied depending on the condition, sex of sibling(s), and provider comfortability ordering testing for family members.

### 3.8. Specialty Clinics

Most participants with a child that screened positive for X-ALD and a few participants with a child who screened positive for Pompe shared that they pursued care at a specialty center after their initial visit with a genetic counselor and other providers at a local facility. Information shared about specialty centers varied. Some families received information from advocacy organizations, other families were given information from providers, and a few families received pushback from providers on referring their child(ren) to a specialty center.

*“So [local clinic] told us they’re, you know, specialist if you want to travel, or you could just do your care locally…As a parent you’re like, local care like obviously. I don’t want to pay for travel. But I also…after that 1st visit I was like with a neurologist locally, they were like, ‘Oh, I’ve never had a patient with ALD’…you don’t want to hear that. You just don’t.” *

*(A3)*

Participants shared that their motivation for seeking care at a specialty center included lack of experience with condition at local center, recommendations from other parents in support groups, and willingness to travel to the center. While some participants found follow-up care at local facilities adequate, other participants shared concerns of limited experience with condition, longer wait times, and underfunding.

*“If [local clinic] had the resources to show us that they could do what we need them to do then I would be comfortable taking him there full time. But it’s not there yet. So it just feels like it’s underfunded there, which is really unfortunate.” *

*(B7)*

*“But when we called [specialty clinic], it was probably, you know, a matter of like a week or 2 that they were able to get us in so that it was kind of funny, because that was faster then the person we could see locally…If I had to do over again, too, I would also have reached out to [specialty clinic] way earlier. I think I felt like I would get better answers from someone locally. And and our local physician knows a lot about ALD. So it’s not that; it’s just not quite as much as the specialists.” *

*(A4)*

Many participants shared that hearing other families’ experiences with specialty centers and advocacy organizations’ perspectives encouraged them to seek out an appointment or continue care for their child at these centers.

Communication

### 3.9. Communication Between Providers and Families

There were comments of parents being satisfied as well as parents being frustrated in their ability to communicate with their child’s providers, including genetic counselors and specialists.

*“But then I knew that essentially that [child] had Pompe, because they just scheduled an appointment and didn’t tell us…so all of a sudden they see it show up in my MyChart that he has a genetics appointment…And so no one called us. No one told us. I think eventually they called us, and said, he has this appointment, but, like that, was it. It was just not good communication at all.” *

*(B7)*

*“She also stapled her contact information on top of the folder like it was right there, front and center. If you have questions, call and or email like this is, this is where I’m at. I will try to get back to you as soon as possible.” *

*(B2)*

Regardless of their family’s personal experience with provider communication, majority of parents shared the importance of being able to contact their child’s care team and the impact that this has on the family feeling supported and heard.

### 3.10. Communication Between Providers

In addition to sharing their families experience with communication between parents and their child’s care team, parents also shared the impact that communication between providers of different specialties had on their family’s experience. Given that the conditions included in this study can involve multiple referrals to assess multiple body systems, referrals to other specialists before or after confirmatory genetic testing is very common. An example of a parent’s experience is quoted below:

*“The geneticist is not communicating to the neurologist or the endocrinologist why my boys are being referred. He sends the referral. I go to the appointment and I get asked, ‘Why are you here?’…I barely know what ALD is. I don’t even really know why I’m here, you tell me. But then, like having to explain, my son has ALD. I was just told by our geneticist to come here.” *

*(A1)*

*“[The pediatrician said] ‘I know what this is about,’ because she got a fax from [the hospital] but she told us like something completely different…she was much more optimistic, I would say, than [the specialist]. And so we kind of sat with these 2 conflicting diagnoses, so to speak, like for the first 3 to 4 weeks of her life because we didn’t go in to see the geneticist and his team until, like she was a month old about.” *

*(A5)*

Parents shared that communication between the multiple providers receiving the newborn screening as well as communication coordinating appointments between specialties impacted their families’ experiences during the results disclosure and also impacted the level of ease coordinating their child’s care.

Holistic Support after NBS

In addition to resources directly related to care, parents expressed the positive impact other types of support had on their families experience as well as types of resources they would have wanted during their journey after their child’s newborn screening result. There was a recurring theme of wanting more holistic resources shared, including advocacy organization information and postpartum mental health support.

### 3.11. Advocacy Organizations and Connection with Other Families

Most of the parents interviewed shared that they are involved in an advocacy organization and/or support group. The way families found organizations and other family connections varied from self-searching to information shared by a provider, with the most common response being the parent seeking out information for groups on their own through social media or internet searches.

*“Between appointments, there’s not much support—I had to ask about advocacy groups…they were very helpful in giving me them when I asked but it wasn’t something that was offered up.” *

*(A5)*

*“So when we found out about it, like, right away, I searched on Facebook to try and see you know who else has it and whatnot. So that’s yeah. That’s how I found this was the mom’s group on for these kids.” *

*(B5)*

There were many different motivations for parents being a part of the advocacy organization or support group. Reasoning included connection with other families, information about treatment and clinical trials, and support navigating care for their child(ren).

*“I mean social media certainly -it’s a blessing and a curse, as we all know. But it definitely made a huge difference in regards to the information provided, and the decrease of panic.” *

*(B3)*

When participants who found groups on their own were asked if they would have liked this information earlier, many shared that even if a parent is not interested or ready to join these groups and organizations, it would be helpful for providers to share this information when speaking with families.

*“We always teach the students like about support groups, and how you should, you know, encourage your patients to do them. But until I lived something where I really needed one, I did not understand how impactful it is to talk to someone else who’s going through that same experience…I don’t know how a genetic counselor could have known what kind of support group to send me to, because it’s so specialized…at that point I had already found them on my own…I think having the support groups to give out would be really helpful. Or just even the organizations that could lead you in the right direction.” *

*(A2)*

*“I wish we would have been [given advocacy organization information] but we were not. I remember, weeks into all of this, after his diagnosis had been confirmed, and such, I remember laying in bed one night not sleeping…And suddenly it dawned on me that certainly we were not the only ones going through something like this. And so then I started looking into advocacy groups and support groups on social media…And we felt very alone up until that point. And then suddenly, it was having access to these lived experiences of other families going through something similar that it was both comforting and informative, and really impacted our experience from that point on.” *

*(B1)*

A few families within the ALD community also shared their experience with more specialized support groups within the organization, such as a group specific for newborn screening or a group specific for boys with X-ALD. While most parents shared that they are involved advocacy organizations, not every participant has sought out this type of resource. A few participants shared hesitation joining specific groups given the difference in their child’s prognosis, such as a child having a different prognosis with early implementation of more recently available treatments for SMA.

*“We were told about CureSMA, and I think we looked at the website a little bit and learned about like the types and the history and stuff, but like didn’t reach out and connect with anyone… I’ve been curious, too, about how it is within the SMA community, between like teens and older adults living with it versus like the babies that are getting these amazing treatments.” *

*(C1)*

### 3.12. Postpartum

Majority of participants noted the impact that postpartum experience had while going through this newborn screening and diagnostic journey. These comments included parents acknowledging the combination of the vulnerable state that an individual is in after giving birth with the emotional toll that a diagnosis after newborn screening can have on a parent.

*“By the time I went in for my 6 week visit, I was like in the danger zone of that postnatal score. I was immediately referred to a psychiatrist and to get help, kind of grieving with and like working through like and coping skills really, because I’m like grieving the loss of someone who’s still alive… I never understood, you know, how there was a mental health crisis in the country. And now I do, sitting where I’m at today.” *

*(A3)*

There were numerous statements of mothers in these interviews wishing that providers took a moment to check in with the birthing parent at appointments for their newborn. Parents also mentioned wanting more opportunities to receive postpartum resources, emphasizing that while not everyone may want or use these resources, it would have been helpful to have the information if the parent was seeking help.

*“Make it a point as a genetic counselor to look mom in the eye and just say, ‘How are you doing?’ Because hormones, right, and postpartum…I would make it a point to say, ‘Mom, how are you? Do you need anything?’” *

*(B2)*

*“The birthing parent is postpartum and like still healing… I was like in a lot of pain in an uncomfy chair, having these like long, hard conversations and stuff…but I think more compassion for the mom and any like support or resources, like maybe extra ones, for the birthing parent.” *

*(C1)*

While not directly related to genetic counseling, there was a recurring theme in parent interviews of seeking postpartum resources and the impact of checking in on the parents’ physical and mental well-being.

## 4. Discussion

**Figure 1:**
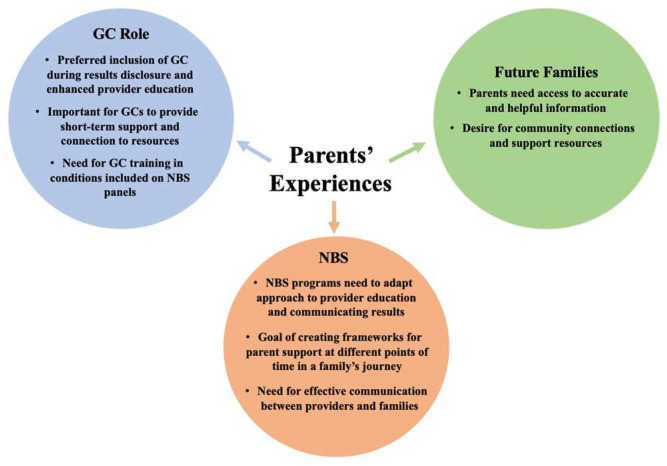
Key Stakeholders in Parents’ Experiences. This figure highlights the impact of using parental experiences to enact change within the newborn screening space. Examples of each stakeholder’s role, framework, or benefit are addressed based on data collected in this qualitative study.

The findings of this study reaffirm the individuality of each family’s experience after a positive newborn screen while also identifying specific points in their journey that could be improved for future families. The goal of this study was to recognize opportunities during the newborn screening process to better support parents, such as communication during the results disclosure or timing of speaking with specialty providers. The messages of this data are applicable to three main stake holders: genetic counselors, newborn screening programs, and rare disease communities and organizations that support these communities, including advocacy organizations. The impact of using parents’ experiences, as depicted in Figure 1, includes addressing the evolving role of GCs in this space, implementing change for newborn screening workflows, and better supporting future families. The relevant messages for each stakeholder are further addressed through following discussion of this research’s results.

### 4.1. Role of Genetic Counselors in Newborn Screening

With the role of genetic counselors in the newborn screening space evolving, this study’s findings highlight potential benefit of involving GCs or genetic specialists during newborn screening results disclosure. Most newborn screening programs follow a model of results being communicated by state’s health department to pediatrician or primary care office, who then shares the results with the family. After this initial phone call, appointments with specialists are typically scheduled. In terms of timing for speaking with a genetic counselor, parents interviewed in this study wanted to speak a genetic counselor as soon as possible, with many parents stating that the wait times increased anxiety or induced panic. With a major motivation for speaking to GCs sooner being accurate information and an opportunity to ask questions, including genetic counselors in the initial results disclosure could help reduce anxiety linked to lack of information or inability to speak with a provider that is familiar with the condition. This recommendation has been echoed in other research, with a 2019 study that explored parental experiences with a positive X-ALD newborn screening including parents’ preference for more provider education and/or a specialist with more background on the condition present on the call [17]. While it would be ideal for every NBS program to utilize genetic counselors in this way, it is also important to recognize the GC workforce limitations, with a small percentage of genetic counselors involved solely with newborn screening programs. Additional data is needed to address the feasibility of incorporating GCs into abnormal newborn screening results disclosures, especially for recently added conditions in each state.

As a result of current workforce constraints and scope-of-practice limitations, widespread integration of genetic counselors in the initial phone call may not be feasible in every state. Taking into consideration the finite number of genetic counselors and recognizing the current practice of non-genetic providers involved in results disclosure, there is potential for GCs to become more involved in provider education as well as reviewing educational materials for providers involved in results disclosure and materials for families. While it is a commonplace practice in multiple states’ infrastructures for GCs working for the state health department to communicate initial screening results to primary care providers, parental experiences in this study reflect that outdated, inaccurate, or unbalanced information was frequently shared with families by non-genetics providers. Additionally, with expansion of newborn screening panels and advancements in the screening technology, knowledge of interpreting complex genomic results and training in communicating these findings to both providers and families is needed in this space. The potential complexity of newborn screening results also emphasizes the importance of adapting information in the context of the presence or absence of symptoms and/or family history.

Given the prevalence of rare genetic conditions and the fact that the conditions studied were more recently added to states’ newborn screening panels, not every provider may have prior experience discussing the natural history of these conditions or specifics of the screening. This emphasizes the role that GCs can play in preparing non-genetics providers for results disclosures in addition to family-friendly resources these providers can share for accurate information on the condition. More data is needed on GCs’ experiences communicating with and educating providers to create more specific recommendations on preparing providers to support families in this space if a GC is unable to share results.

### 4.2. Adapting Newborn Screening Programs

In response to the evolving role of genetic counselors in newborn screening as well as conditions being added to the RUSP, parental experiences can be used to recognize opportunities for improvement in the newborn screening process. This data emphasizes genetic counselors’ roles but also raises concerns about larger issues, such as shortcomings in communication and follow-up support for families after a newborn screen results disclosure. An additional research opportunity to build upon the data collected in this study could be exploring how NBS education is currently incorporated into the curriculum of healthcare professionals’ training programs.

As newborn screening programs expand to include additional conditions, state programs must ensure that parent support is a priority in all three of the timeframes (newborn screening, diagnostic, and follow-up). Parent voices echoed a need for change, and poses the important question of who can fill this role? If NBS programs are going to evolve their communication systems and follow-up, who is responsible for addressing these changes? There was an emphasis in many interviews that parents wanted an individual able to answer questions giving the results call or available at the time of the results disclosure. GCs’ training in both rare genetic conditions and psychosocial counseling make it a possibility for newborn screening programs to increase the number of genetic counselors in these roles. As previously mentioned, it is important to explore the potential role of genetic counselors bridging the gaps in support during the newborn screening process while also acknowledging the limitations in workforce bandwidth.

While not every state has a robust team of GCs that could manage every positive newborn screen results disclosure, state newborn screening programs have implemented different frameworks to increase earlier access to a genetic counselor and provide parents with the opportunity to ask questions after a NBS results disclosure. Minnesota Department of Health’s Newborn Screening Program has an on-call genetic counselor for after-hour consultations [18]. Iowa’s newborn screening protocol includes a family contact program that calls the family after the primary care provider has shared the results, providing the family with an opportunity to ask questions or clarify next steps [19]. While no parent in this study explicitly mentioned these specific states’ short-term follow-up programs, the success of these state NBS programs’ incorporation of GCs can serve as an example for states looking to evolve their own programs, which offers an opportunity for communication amongst NBS staff in different states and potentially other nations. In order to implement change and justify funding for other states’ newborn screening program as many NBS programs do not currently have the resources or funds to implement these specifically mentioned models, additional data is needed on current workflows and resources that have proven beneficial at that state’s level. Another research opportunity exists in further exploring the relationship between parent satisfaction rates with the NBS process after a positive/abnormal result and the state in which the child was born. The utilization of genetic counselors in short-term follow-up after a newborn screening result can be a potential first step to addressing increased support and accessibility to information for families prior to the initial genetic counseling appointment.

As briefly mentioned in the discussion, many gaps in the newborn screening infrastructure are systemic—impacted by factors such as limited resources and variability between state programs. Participant interviews as well as previous state newborn screening projects have emphasized the value of genetic counselors in newborn screening. The implications of these communication and support shortcomings within the newborn screening process on parental experiences can be used to advocate for change in the system’s workflow, including funding more support for genetic counselors or other providers in this space who can provide support and improve communication. While this study highlighted the potential involvement of genetic counselors as part of the solution, additional trainings for providers, state funding, and broader reform in the newborn screening space is needed.

### 4.3. Support for Parents During Newborn Screening and Diagnosis

Parent preferences on most helpful and least helpful information during the initial genetic counseling session varied from family to family leading to an overall recommendation of GCs meeting the family where they are at and exploring what information is most important to them in that initial session. The recurring theme of finding handout information and parent-friendly resources on the condition aligns with previous research exploring helpful information and parents’ experiences with other genetic conditions on NBS. A 2013 study that focused on families’ experiences with a positive cystic fibrosis (CF) NBS found that over 50% of the participants in this study mentioned written or printed out materials for families as a way to improve the newborn screening experience [20]

Parents’ also desire community support during their experience after a positive newborn screen. GCs’ role of supporting families in this space has the potential to extend past the bounds of genetics and clinical care of the child, using the session as an opportunity to check in on family members and provide more holistic types of resources. The parental experiences shared in this study encourage a more holistic approach to supporting families that involves access to advocacy organizations and mental health resources along with accurate information about the condition their child screened positive for on newborn screening.

In addition to this study’s unexpected but important theme of participants wanting support displayed for the postpartum mother, other studies have explored the role for anxiety and stress in parents of children with rare disease. A 2022 study addressing psychosocial concerns with newborn screening and highlights the adverse impact that NBS result-induced anxiety can have on parents when interacting with their child [21]. With 1 in 7 women experiencing perinatal depression [22], it is crucial that all parents, especially parents within the rare disease community, receive support postpartum. There is a need for genetic counselors to use genetic counseling sessions as a space to check-in and explore resources that the family is seeking emphasizes the need to at least present this type of information to parents. While long-term support psychosocial and resource support is more appropriately managed by other providers, such as psychologists, psychiatrists, and social workers, genetic counselors can serve as a conduit of care and play a role in connecting families with the resources they are seeking. Genetic counselors’ knowledge of available resources and collaboration with other professionals can help support parents in their newborn screening journey.

In addition to the findings shared in the results section and recommendations discussed in this section, representative participant quotes shared in the interview when asked about advice for both genetic counselors and future families going through a diagnosis after newborn screening can be found listed in Figure S1 and Figure S2, respectively, in the Supplementary Materials section of this article. The advice that parent’s shared through this study is invaluable information that can be used by genetic counselors in the newborn screening space, as well as other providers, to tailor their approach in this setting and utilize the shared experiences that families on this journey have found helpful.

### 4.4. Limitations

Limitations of this study include lack of diversity in participant’s demographic information, including diversity in race, education, maternal vs. paternal perspective, and involvement in advocacy organizations. All participants identified as White or Caucasian and majority obtaining a form of higher education past high school. Also, given all participants were birthing mothers, this study does not include the voices and unique experiences of fathers after a child’s positive newborn screen. Given the recruitment methods, voices of parents that are not active in advocacy groups may not be reflected in this study’s data. The goal for recruitment for each condition was 5 to 10 parents. This was not reached for the SMA group, and thus, condition specific experiences could not be compared in this sub-group. The aspects of the participant demographics and recruitment methods mentioned in this section may limit the generalizability of the findings while encouraging additional research expanding participant diversity in future studies.

### 4.5. Future Directions

Because this study looked at multiple conditions and had a small sample size for each condition, future research opportunities have the potential to focus specifically on one condition to further analyze disease-specific parent preferences. Additional research studies that focus on a diverse sample involving different states’ newborn screening programs could provide insight on state level variation and the impact on parental experiences. There has been research conducted on a state level to evaluate newborn screening workflows in the state, which provides an opportunity for future research or collaboration across state newborn screening programs. Implementing existing workflows in newborn screening programs that have been successful in other states is possible but would require program resources and likely funding in order to support these implementations. Another area that could be further researched is the impact of available treatments that improve prognosis on the desire for community support. For example, some parents may want to join specific groups for the treatment their child is on while others may be hesitant to join a support group if their child has a different prognosis with the administration of the available treatment; the latter was mentioned by one participant in this research. This direction of research could help providers offer appropriate support resources as well as inform organizations on ways to structure sub-groups within their community, such as by age of child, treatment regimen, or clinical versus NBS diagnosis.

As newborn screening expands, the conversation around genetic counselors’ role in the process will continue to happen, and research in other countries have highlighted the impact of GCs in this space. A recent study conducted in Australia compared and contrasted experiences in a cohort of parents who had access to a GC after a positive NBS for SMA and a cohort of parents who did not have access to a GC after the same NBS result; the results of this research align with this article’s finding of the impact that the provider’s knowledge of the condition and access to a GC can have on parents’ experiences during the time of the results disclosure [23]. It is important to note that the research methods used and the context of the studies differ between this paper’s study and Australian study. However, the findings of both emphasize the need for more comparative data in future research that explore parental experiences at an international level regarding newborn screening protocols and workflows to elicit geographic, cultural, or soft infrastructural differences impacting families’ perspectives.

## 5. Conclusions

This study highlights the diverse parental experiences with their child’s diagnosis after a true positive newborn screening. Key findings suggest that involvement of GCs or genetics providers earlier in the process, such as during the results disclosure, could mitigate families’ anxiety as well as serve as a resource for accurate and up-to-date information on the condition their child screened positive for. This study is not without limitations, including small sample size and lack of diversity among participants, which emphasize the need for additional research on parental preferences. Opportunities for greater depth on condition-specific preferences is also needed for recommendations that can best support each rare disease community’s needs. Addressing the areas that newborn screening can be improved and adapting the role that genetic counselors serve in this space can contribute to better experiences for future families going through a diagnosis after newborn screening.

## Figures and Tables

**Table 1:** Demographic information of the participants in the study.

Code	Condition	Highest Level of Education	Household Income	Ethnicity
A1	X-ALD	Some college, but no degree	$25,000– $49,999	White or Caucasian
A2	X-ALD	Graduate or professional degree (MA, MS, MBA, PhD, JD, MD, DDS)	$100,000– $149,999	White or Caucasian
A3	X-ALD	Graduate or professional degree (MA, MS, MBA, PhD, JD, MD, DDS)	$150,000 or more	White or Caucasian
A4	X-ALD	Bachelor’s degree	$150,000 or more	White or Caucasian
A5	X-ALD	Graduate or professional degree (MA, MS, MBA, PhD, JD, MD, DDS)	$150,000 or more	White or Caucasian
B1	Pompe	Graduate or professional degree (MA, MS, MBA, PhD, JD, MD, DDS)	$150,000 or more	White or Caucasian
B2	Pompe	Some college, but no degree	$100,000 –$149,999	White or Caucasian
B3	Pompe	Graduate or professional degree (MA, MS, MBA, PhD, JD, MD, DDS)	$150,000 or more	White or Caucasian
B4	Pompe	Bachelor’s degree	$150,000 or more	White or Caucasian
B5	Pompe	Associates or technical degree	$75,000 –$99,999	White or Caucasian
B6	Pompe	Graduate or professional degree (MA, MS, MBA, PhD, JD, MD, DDS)	$100,000 –$149,999	White or Caucasian
B7	Pompe	Graduate or professional degree (MA, MS, MBA, PhD, JD, MD, DDS)	$100,000 –$149,999	White or Caucasian
C1	SMA	Graduate or professional degree (MA, MS, MBA, PhD, JD, MD, DDS)	$100,000 -$149,999	White or Caucasian

**Table 2:** Themes and subthemes from parent interviews.

Theme	Subthemes
NBS/Results Disclosure	3.1 NBS workflow 3.2 Parent’s knowledge of NBS 3.3 Impact of results disclosure
Diagnostic Process after NBS/Prognostic Journey	3.4 Scheduling/timing of initial GC appointment 3.5 Information during initial GC appointment 3.6 First family’s experience
Treatment/Follow-Up	3.7 Testing family members 3.8 Specialty clinics
Communication	3.9 Communication between providers and families 3.10 Communication between providers
Holistic Support after NBS	3.11 Advocacy organizations and connections with other families 3.12 Postpartum

## Data Availability

Due to privacy concerns and institutional policies protecting participant identifiability, raw data that is linked to participants’ identities is not available from this study. Non-identifying data from this research is included in the article/Supplementary Materials.

## Supplementary Materials

The following supporting information can be downloaded at: https://www.mdpi.com/article/10.3390/ijns11040101/s1, Table S1: Additional Representative Participant Quotes; Figure S1: Participant Quotes of Advice for Genetic Counselors; Figure S2: Participant Quotes of Advice for Other Parents.
