# Supplementary material for: A Qualitative Study on Parental Experiences with Genetic Counseling After a Positive Newborn Screen for Recently Added Conditions on the Recommended Uniform Screening Panel (RUSP)
Source: Int J Neonatal Screen. 2025 Oct 30;11(4):101. doi: 10.3390/ijns11040101 (PMC12641915; doi:10.3390/ijns11040101)
Supplement: Supplementary file 1 [file IJNS-11-00101-s001.zip › IJNS-3817557-supplementary.pdf]

International Journal of Neonatal Screening

MDPI

Supplementary Materials

Table S1. Additional Representative Participant Quotes

Theme	Subthemes
NBS / Results Disclosure	**3.1 NBS workflow**<br>*"It seemed like my son's pediatrician had more of a knowledge as to what exactly was going wrong with the newborn screening and why we were getting told over and over again is inconclusive. I wish she would have maybe communicated that with me. Now, I understand she probably didn't want put me in a state of panic, but at the same time I was getting angry…I don't want to keep testing him over and over. This is ridiculous… so I wish they would have communicated a little bit better" (A1)*<br>**3.2 Parent's knowledge of NBS**<br>*"I don't think I knew much about the screening before having them…I remember them taking like the blood for the sample. And they're like, Oh, yeah, we just do this state newborn screening whatever. So I didn't think much of it and didn't think anything was going to come back from it." (C1)*<br>*"The nurse came in and was like, Hey, this, saying we're gonna prick his heel. You know, if you don't hear from the hospital, no news is good news, and I remember that quote so vividly, and I didn't think anything about it after that, because again, like, there's no genetic history of disorder on either side of our families, I had a completely healthy pregnancy." (B6)*<br>**3.3 Impact of results disclosure**<br>*"[The pediatrician] tried their best, but [not] being overly familiar, you know they know the bare minimum…he reassured, and again mentioned the fact that the enzyme could be low if she was a carrier but past that nobody knew what the next step was of where to go." (B3)*<br>*"[The provider] also told me like, don't go on the Internet and look this up…just wait for the result. But obviously, like, I'm gonna go on the Internet. I don't know what this is and if my son might have it. So of course, I went on the Internet, and you know, learn more about it." (A1)*<br>*"If somebody who could have answered my questions had given me the phone call, it would have saved us days of complete peril…when we got to the genetic counselor she said to us, like, I didn't want to panic you by calling myself. and I was like, no, I really wish you had, because it was so much more panic not hearing from you." (A2)*
Diagnostic Process after NBS /Prognostic Journey	**3.4 Scheduling/timing of initial GC appointment**<br>*"Luckily, it's only like an hour. And we have family around that was more than willing to watch her. The only thing that, like I could really think about was that the reason I think it was scheduled for Wednesday was that the genetic counselor was on vacation." (B4)*<br>**3.5 Information during initial GC appointment**<br>*"The geneticist told us that there was a good chance that [child] was either going to have late onset, was either a carrier for Pompe, or was going to have very late onset, and this was something we weren't going to have to worry about until he was, you know, a teenager or adult. I felt that that was misleading…They couldn't tell me how they would know that…I didn't appreciate the speculation…I know that as parents, we're always going to push for more information…But I wouldn't want them to just fill the void…I would have appreciated a straightforward response that we truly don't know" (B1)*<br>*"It was very helpful to hear from genetics that you know…that even if we did have more children who were affected with Pompe disease, they would likely also be late onset, and they would likely also not have a very severe presentation of it….That the odds are much more in our favor to have children who are unaffected than affected. So that was super reassuring." (B6)*<br>**3.6 First family's experience**<br>*"She was the first baby in {state} that was positive…And so they didn't know what to tell us to do about extended family." (A2)*
Treatment / Follow-Up	**3.7 Testing family members**<br>*"So after her diagnosis, we were able to cheek swab her older siblings, and she does have 2 brothers with late onset." (B2)*<br>*"We both got tested, and then my negative result came back a day before his positive. It was like restarting the grief process over again when we realized it was [child's father]." (A2)*<br>**3.8 Specialty clinics**

	"If I had to do over again, too, I would also have reached out to {state} way earlier. I think I felt like I would get better answers from someone locally. And our local physician knows a lot about ALD. So it's not that; it's just not quite as much as the specialists." (A4)<br>*"It'd be nice to know that they exist, especially like depending on who you're working with geographically." (A3)*
**Communication**	**3.9 Communication between providers and families**<br>*"I don't even have my local doctor's email, I don't even know how to get a hold of him if something urgent comes up and so I feel like that's really difficult, too, when you have one of these rare diseases, because things happen with kids. And you're like, oh, no! What do we do like? And so it's so nice to have somebody so readily available." (A4)*<br>**3.10 Communication between providers**<br>*"[The genetic counselor] said, 'what did your pediatrician tell you?' And so I told her, and she just like shook her head because she was like, I didn't say any of that. And then going back and googling things. I'm like well, she clearly just googled it and that's what she told me." (B4)*
**Holistic Support after NBS**	**3.11 Advocacy organizations and connections with other families**<br>*"It was really helpful going and finding like a Facebook group for moms of kids with Pompe, because obviously, like mom…they're all going through it…You care the most about your kids. So it's like, you're gonna like, look at everything and they just have like a lot of advice, a lot of support." (B4)*<br>*"It's through these groups that I find out about, like the clinical trials that are going on." (A5)*<br>**3.12 Postpartum**<br>*"Our hormones alone put us at high vulnerability. So then, to drop something like this, and I'm very fortunate that I work in healthcare, and I can advocate, and I chased for follow-ups, and I knew who to go for what. And I have contact points. But someone somewhere just needs to help do better in regards to maybe families who don't have those resources because mom alone is already dealing with her postpartum." (B3)*

Figure S1. Participant Quotes of Advice for Genetic Counselors

The following quotes are parents' advice for genetic counselors when counseling families in the process of a diagnosis after a positive newborn screen result. The interview question specifically meniotned specifically mentioned GCs; however, some of the advice below is applicable to other healthcare providers involved in the newborn screening process.

- *"Let them have their moment after that diagnosis is given…I would just feel like let them, if they're going to have a moment in in the office or in the appointment, just let them have that moment. [Ask] 'what do you need from me at this point?... What do you need? What you know? Do you need a minute?'" (B2)*
- *"When you're delivering this news to people who are…at a risk of postpartum anxiety or depression, like adding that tidbit of hope at that initial appointment would mean the world. It would have changed the way I had handled that 1st year." (A3)*
- *"Just to be really patient with them. Because it's all new. It's overwhelming. To really just explain things in ways that we can understand cause like I said, we're not doing this every day…being reassuring, not rushing." (B4)*
- *I think empathy is a huge thing…these are potentially very life altering…news that you can get like for your child for potentially the rest of your family. My whole family was kind of on pins and needles…setting expectations, being as upfront as possible is helpful you know. Not sugar coating it." (A2)*
- *"Be empathetic. and like, listen. Hear people out, whatever they're fears and concerns are…Have a balanced approach that's not like overly optimistic, but obviously not fear mongering either." (C1)*
- *"Maybe just instead of focusing on…the what ifs, you know, try to find a way to focus on the positives and the plan." (B3)*

Figure S2. Participant Quotes of Advice for Other Parents

The following quotes are parents' advice for future families going through a diagnosis after a positive newborn screen, with some quotes being more specific to advice about the genetic counseling appointment and other quotes being more general to the newborn screening and diagnosis journey.

- *"Taking it day by day, 'cause it feels so overwhelming." (A4)*
- *"Give yourself, as parents, grace…lean into your support system, your faith, your family, your friends." (B2)*
- *"Get help if you need it. And find people who can relate and you can ask questions to that aren't always providers." (A3)*
- *"Definitely try and reach out and find other parents or people who are going through something similar." (B4)*
- *"Take it minute by minute, and just still trying to just love on that little baby. I think that's probably the biggest thing. This is easy to forget that." (B7)*
- *"Write down all your questions to bring in to the genetic appointment with you…it's hard to remember everything they said and remember all the questions you had." (A2)*
- *"I think it's really important for these parents or caregivers to identify…who their team is, who their contact person is. Every health system as you said, earlier, handles things differently." (B3)*
- *"It would be learn as much as you can, and don't panic because…it is really hard, it is really difficult, it is scary, and it is uncertain. But it doesn't have to be the end of the world, and it's your job as much as it is to advocate for them to give them a sense of normalcy, and that begins with you having a sense of normalcy." (B6)*
